# Exploring the Interplay between Asthma and Hemoglobinopathies: A Comprehensive Review

**DOI:** 10.3390/jcm13113263

**Published:** 2024-05-31

**Authors:** Cristiana Indolfi, Giulio Dinardo, Carolina Grella, Angela Klain, Alessandra Perrotta, Gianluca Mondillo, Maria Maddalena Marrapodi, Fabio Decimo, Michele Miraglia del Giudice

**Affiliations:** Department of Woman, Child and of General and Specialized Surgery, University of Campania Luigi Vanvitelli, 80138 Naples, Italy; cristianaind@hotmail.com (C.I.); carolina.grella@studenti.unicampania.it (C.G.); klainangela95@gmail.com (A.K.); alessandra.perrotta@studenti.unicampania.it (A.P.); gianluca.mondillo@studenti.unicampania.it (G.M.); mariamaddalena.marrapodi@unicampania.it (M.M.M.); fabio.decimo@unicampania.it (F.D.); michele.miragliadelgiudice@unicampania.it (M.M.d.G.)

**Keywords:** asthma, hemoglobinopathies, sickle cell disease, thalassemia, children, cytokines, pediatrics, asthma management, spirometry, asthma screening

## Abstract

Asthma, a prevalent chronic respiratory condition characterized by inflammation of the airways and bronchoconstriction, has demonstrated a potential association with hemoglobinopathies such as thalassemia and sickle cell disease (SCD). Numerous studies have highlighted a higher prevalence of asthma among thalassemia patients compared to the general population, with rates ranging around 30%. Similarly, asthma frequently coexists with SCD, affecting approximately 20–48% of patients. Children with SCD often experience heightened lower airway obstruction and airway hyper-reactivity. Notably, the presence of asthma in SCD exacerbates respiratory symptoms and increases the risk of severe complications like acute chest syndrome, stroke, vaso-occlusive episodes, and early mortality. Several studies have noted a decrease in various cytokines such as IFN-γ and IL-10, along with higher levels of both IL-6 and IL-8, suggesting an overactivation of pro-inflammatory mechanisms in patients with hemoglobinopathies, which could trigger inflammatory conditions such as asthma. The exact mechanisms driving this association are better elucidated but may involve factors such as chronic inflammation, oxidative stress, and immune dysregulation associated with thalassemia-related complications like chronic hemolytic anemia and iron overload. This review aims to comprehensively analyze the relationship between asthma and hemoglobinopathies, with a focus on thalassemia and SCD. It emphasizes the importance of interdisciplinary collaboration among pulmonologists, hematologists, and other healthcare professionals to effectively manage this complex interplay. Understanding this link is crucial for improving care and outcomes in affected individuals.

## 1. Introduction

Asthma is a chronic inflammatory disease of the airways, where different immune system cells, both innate and adaptive, work together with epithelial cells to cause bronchial hyper-reactivity, resulting in increased sensitivity of smooth muscle cells. Asthma ranks as the most prevalent chronic condition in childhood, affecting almost 1 in 10 children and 1 in 12 adults, resulting in significant morbidity and substantial healthcare costs worldwide [1,2,3]. Traditionally, asthma has been classified into two clinical forms [4]. The majority of children and approximately 50% of adults experience allergic asthma, characterized by allergic sensitization marked by elevated serum immunoglobulin E (IgE) antibodies and positive skin-prick tests to common allergens like house dust mites and various pollens or food allergens [5,6]. Allergic asthma is traditionally viewed as stemming from inflammation triggered by aeroallergens, mediated by T-helper type 2 (Th2) responses and cytokines such as IL-4, IL-5, and IL-13. IL-13, produced by various immune cells, is considered a key player in asthma-related inflammation [3]. On the other hand, nonallergic (intrinsic) asthma typically develops later in life, lacking IgE reactivity to allergens and evident involvement of the adaptive immune system, such as type 2 helper T cells (Th2 cells) [5,6]. Non-allergic asthma is characterized by neutrophilic or paucigranulocytic airway inflammation, primarily supported by IL-8, IL-17, IL-22, other T cell-related cytokines, and cytokines derived from epithelial cells [3,7]. Asthma is characterized by a history of respiratory symptoms, which encompass wheezing or recurring coughing, shortness of breath, and a sensation of tightness in the chest accompanied by pronounced variations in expiratory airflow [8]. Recent updates in guidelines stress the importance of a thorough evaluation of symptoms, including nocturnal occurrences, limitations in daily activities, and a patient’s history of exacerbations, to ensure accurate diagnosis and effective management of asthma [9]. Furthermore, the incorporation of biomarkers such as fractional exhaled nitric oxide (FeNO) and eosinophil counts in blood or sputum aids in refining the classification of asthma subtypes and facilitates the implementation of tailored therapeutic approaches for individual patients [10,11,12,13]. The efficient transportation of oxygen in red blood cells relies on hemoglobin, a tetrameric protein made up of two α-globin and two β-globin chains. Genetic abnormalities can lead to hemoglobinopathies, including sickle cell disease (SCD) and thalassemia, which are characterized by abnormal hemoglobin variants or reduced hemoglobin levels [14]. SCD results from a single amino acid substitution in the β-globin chain, forming hemoglobin S (HbS) and causing red blood cells to sickle under low-oxygen conditions. This abnormal cell morphology triggers a series of complications, including chronic hemolytic anemia and vaso-occlusive crises (VOC), which can manifest as acute chest syndrome (ACS), pulmonary hypertension, and pulmonary fibrosis [15,16]. Thalassemia involves deficient production of α- or β-globin chains due to genetic mutations. α-thalassemia results from deletions or mutations in the α-globin genes, while β-thalassemia arises from mutations in the β-globin gene, with clinical severity varying from asymptomatic to hemolytic anemias and severe transfusion-dependent anemias [17,18]. Several studies suggest a potential association between inflammatory atopic diseases like asthma and anemia in pediatrics, particularly concerning asthma and hemoglobinopathies [14,18]. However, the specific interplay between asthma and hemoglobinopathies, particularly thalassemia and SCD, remains to be fully elucidated [19]. Chronic inflammation is a hallmark of both asthma and hemoglobinopathies. Furthermore, hemoglobin variants, such as hemoglobin S in sickle cell disease, may exert direct effects on pulmonary vascular tone and endothelial function, potentially exacerbating bronchial hyper-reactivity and airway obstruction in asthmatic individuals with hemoglobinopathies. Additionally, the comorbidities associated with hemoglobinopathies, such as chronic anemia and recurrent vaso-occlusive crises, could contribute to the complexity of asthma management in affected individuals. The aim of this study was to examine the frequency and relationship between asthma and major hemoglobinopathies and to ascertain whether children with hemoglobinopathies are more likely to have asthma compared to children without hemoglobinopathies. Understanding this intersection is essential for developing comprehensive treatment approaches for pediatric patients with both conditions.

## 2. Asthma Prevalence in Children with Thalassemia

The connection between thalassemia and asthma has been relatively overlooked, particularly concerning children (Table 1). In a study conducted by Hsieh HY, data from a randomly selected sample of one million individuals from the National Health Insurance Research Database were employed to compare a cohort of thalassemia patients with a control group [19]. According to this research, children with thalassemia showed a greater prevalence of asthma compared to those without thalassemia (33.6% vs. 23.2%, *p* < 0.001). Moreover, the results of the study revealed a noteworthy association, suggesting that children with thalassemia were more likely to experience asthma attacks [19]. Rhew K. et al. investigated the possible connection between anemia in pediatric patients and inflammatory atopic illnesses, such as atopic dermatitis, allergic rhinitis, and asthma. A cross-sectional analysis was carried out using a pediatric dataset from the Health Insurance Review and Assessment Service (HIRA) of South Korea from 2016 [20]. A multivariable logistic regression approach was utilized to control for demographic variables and investigate the correlation between iron deficiency anemia (IDA) and atopic disorders. The analysis showed a strong correlation between IDA and atopic illnesses, with patients having multiple atopic conditions displaying higher prevalence rates of IDA, pointing to a possible link between IDA in pediatric patients and atopic illnesses [20]. Additionally, a study conducted by Palma-Carlos examined the prevalence of asthma in people with hemoglobinopathies who had dust mite allergies, with a particular emphasis on thalassemia and sickle cell trait patients [21]. For the study, 4,000 patients from an outpatient allergy clinic over the previous five years were sampled. In this study, it was found that patients with hemoglobinopathies (e.g., beta-thalassemia and sickle cell disease) often had respiratory allergies, with a significant number suffering from asthma. This group showed a higher prevalence of asthma compared to a control group without hemoglobinopathies, suggesting that hemorheological changes might influence bronchial hyperreactivity [21].

## 3. Asthma in Children with Sickle Cell Disease

A diagnosis of asthma has been associated with increased mortality and morbidity in people with SCD [22,23,24]. Individuals affected by SCD encounter both acute and persistent complications stemming from the presence of sickled red blood cells, which obstruct blood vessels and lead to hemolysis, anemia, inflammation, and damage to vital organs. Among these complications, pulmonary issues are prevalent, manifesting as recurring symptoms like wheezing and chest tightness. Medical practitioners often categorize these symptoms as asthma, sleep-related breathing disorders, or various forms of lung disease characterized by obstruction or restriction [22,25]. The incidence of clinician-diagnosed asthma among pediatric SCD patients is notably higher, affecting around 25% (with a range of 20% to 48%) compared to 11% in the general African American youth population in the United States. Asthma-like manifestations, including wheezing, chest tightness, and coughing, are common among pediatric SCD patients, with airway hyperresponsiveness being significantly more prevalent in this group compared to the general population, irrespective of an asthma diagnosis [26]. The underlying mechanisms behind these asthma-like symptoms remain unclear, and there is ongoing debate regarding whether they should be solely attributed to asthma or considered as part of the SCD pathology.

### 3.1. Asthma as a Risk Factor for VOC and ACS in Patients with Sickle Cell Disease 

Nevertheless, pediatric SCD patients diagnosed with asthma face a significantly heightened risk of hospitalization due to acute chest syndrome, severe pain episodes, and a twofold increase in mortality compared to SCD patients without an asthma diagnosis [23]. Findings from various studies shed light on the clinical relationship between SCD and asthma (Table 2). Boyd et al.’s extensive study revealed that children with SCD diagnosed with asthma experienced nearly double the episodes of ACS and VOC compared to those without asthma [22]. Additionally, Knight-Madden et al. discovered a higher likelihood of atopic asthma among SCD children with recurrent ACS episodes [27]. Likewise, repeated ACS episodes are believed to contribute to the development of sickle cell chronic lung disease, as suggested by Hagar et al. [28]. They also found a link between asthma or obstructive lung disease and an increased risk of pulmonary hypertension in SCD children [28]. Indeed, the presence of asthma in individuals with SCD could be an additional risk factor for the health of these patients, as an analysis from a prospective cohort study by the Cooperative Study for SCD revealed that subjects diagnosed with asthma had more than double the mortality risk compared to those without asthma, with a significant difference in median lifespan [25]. In a study by An et al., a comprehensive analysis was conducted on 521 children with SCD, focusing on total IgE levels and the prevalence of asthma. Of these children, 27% were diagnosed with asthma, with a significantly higher prevalence among boys than girls. Notably, total IgE levels were substantially elevated in the SCD population, with around 50% of participants having levels above the 90th percentile compared to the general population. Moreover, elevated total IgE levels were associated with an increased risk of asthma diagnosis, as well as an increased incidence of ACS episodes [29]. Despite previous studies, in a study by Angel et al., aimed at uncovering the prevalence and connection between asthma, allergic sensitization, and modified pulmonary function in individuals diagnosed with SCD of both types (SS and Sβ^0^), it was observed that there were no significant differences in the prevalence of asthma between SCD patients and the control group, with rates of 4.3% and 13.6%, respectively. Furthermore, SCD patients had lower frequencies of wheezing, dry cough at night, and steroid use compared to the control group [30]. 

### 3.2. Screening Strategies for Asthma in Sickle Cell Disease

Given the increased health risk for patients with both asthma and SCD, screening studies have been conducted to assess the prevalence and determine the most effective methodologies. In research by Duckworth et al., it was suggested that there is merit in implementing screening protocols for asthma among individuals diagnosed with sickle cell disease. The aim of the study was to assess the percentage of African American children and young adults aged 5–34 years SCD who underwent spirometry testing and its correlation with a diagnosis of asthma and/or ACS. The results indicated that out of 2749 African American patients identified with SCD, 577 had asthma, and 409 had a history of ACS. Among them, 249 had both SCD and ACS with asthma. Among these patients, 77 underwent spirometry during the study period [31]. Moreover, Sadreameli et al. hypothesized that a screening program could help identify children with SCD who needed a referral to a pulmonary clinic for asthma management. They conducted a single-center project screening SCD patients for asthma using a validated questionnaire and portable spirometry. The study found that 37% of the participants had a positive asthma screening, with a sensitivity of 87.5% and specificity of 85.3%. However, only a small percentage of those referred for further evaluation were seen in the pulmonary clinic. This highlights the need for improved screening and treatment strategies to reduce pulmonary morbidity in SCD [23]. The assessment of asthma presence could also be extended to the preschool population to ensure accurate identification of comorbidities and appropriate treatment. In a study conducted by Chan et al., the researchers aimed to validate the Breathmobile Case Identification Survey (BCIS) for asthma screening in preschool-aged children with SCD. The research involved a prospective, single-center study of 50 children aged 2–5 years with SCD. All patients were administered the BCIS, and a pulmonologist, blinded to the results, evaluated patients for asthma. The BCIS demonstrated high sensitivity, specificity, positive predictive value, and negative predictive value for identifying asthma. The study concluded that the BCIS serves as an effective asthma screening tool in preschool children with SCD, highlighting the low prevalence of asthma in this age group [32].

**Table 2:** Asthma and sickle cell disease.

Reference	Study Design	Objectives	Population	Methods	Results
Boyd, 2006 [22]	Observational cohort study.	Investigated the association between asthma and SCD-related morbidity in children.	291 African American children with SCD.	Prospective data collection through the Cooperative Study of Sickle Cell Disease (CSSCD).	Asthma was associated with increased episodes of ACS and painful episodes in children with SCD; younger age at first ACS episode and increased transfusion requirement in children with SCD and asthma.
Sadreameli, 2017 [23]	Observational cohort study.	Screened pediatric patients with SCD for asthma and pulmonary function abnormalities.	157 pediatric patients with SCD aged 2 to 21 years.	Screening with a validated questionnaire (Breathmobile) and portable spirometry.	58 (37%) screened positive for asthma; 105 (83%) had interpretable spirometry, 35 (34%) abnormal; questionnaire sensitivity 87.5%, specificity 85.3% for clinician-diagnosed asthma.
Boyd, 2007 [25]	Prospective cohort study.	Investigated if asthma is a risk factor for mortality in individuals with SCD.	1963 individuals with SCD.	Participants were assessed for asthma diagnosis and mortality cause.	Asthma was associated with over a two-fold higher risk of mortality after adjusting for known risk factors, resulting in a shorter median lifespan for individuals with asthma compared to those without.
Strunk, 2014 [26]	Prospective cohort study.	Investigated the association between asthma diagnosis and ACS episodes in children with SCD.	Children with SCD.	Questionnaire-based assessment of wheezing symptoms, parental asthma history, lung function tests, allergy skin tests	Wheezing symptoms and parental asthma history were strongly associated with asthma diagnosis in children with SCD.
An, 2011 [29]	Prospective cohort study.	Investigated the association between total and specific IgE levels and asthma diagnosis, as well as their association with ACS and pain episodes in children with SCD.	521 children with SCD.	Serum total and specific IgE levels were assessed, physician-diagnosed asthma was documented, and the incidence rates of ACS and pain episodes were recorded.	Elevated total and specific IgE levels associated with increased risk of asthma diagnosis and ACS episodes elevated IgE levels suggestive as a potential biomarker for asthma risk in children with SCD.
Angel, 2020 [30]	Observational cohort study.	Investigated the association between asthma, allergic sensitization, and lung function in SCD patients.	Children and adolescents with SCD.	The study evaluated laboratory parameters, allergic sensitization, and lung function in SCD patients.	It found that asthma and bronchial hyperreactivity play an important role in SCD patients.
Duckworth, 2020 [31]	Multicenter observational study	The objective of the study was to determine the use of diagnostic spirometry testing in African American children and young adults with SCD with or without asthma and/or ACS.	African American children and young adults aged 5-34 years with SCD, with or without asthma and/or ACS.	Query of electronic medical record data from four geographically diverse academic medical centers.	Among 2749 patients with SCD identified, 577 had asthma and 409 had ACS. Only 31% of patients with SCD, ACS, and asthma received spirometry testing. Significant associations were found between asthma and ACS across all centers.
Chan, 2023 [32]	Prospective, single-center study	The objective of the study to validate the Breathmobile Case Identification Survey (BCIS) as an asthma screening tool in preschool children with SCD.	50 preschool children (aged 2–5 years) with SCD.	Administered BCIS to all patients; pulmonologist evaluation for asthma; obtained demographic, clinical, and laboratory data.	No significant differences in clinical demographics between groups with or without history of ACS.

## 4. Inflammatory Pathways Dysfunction in Hemoglobinopathies and Asthma

Epidemiological data indicate that a significant proportion of patients with hemoglobinopathies also have asthma. Ongoing research is investigating the common inflammatory pathways that might explain this association, as an asthma diagnosis can serve as an early warning sign for an increased risk of developing ACS in individuals with SCD [33]. 

### 4.1. Arginine Metabolism and Pulmonary Issues

A study conducted by Morris et al. reviewed past cases of patients with SCD from various medical centers. They found that dysregulated arginine metabolism, marked by increased arginase activity, plays a key role in causing pulmonary issues in SCD. These metabolic pathways are also important factors in asthma and pulmonary hypertension. The processes underlying both vasculopathy and hemolysis-related pulmonary problems in SCD likely involve similar mechanisms, which could help explain the high occurrence of asthma and pulmonary hypertension. Hemolysis within blood vessels shifts arginine metabolism away from producing nitric oxide (NO), leading to pathways dependent on ornithine. This shift contributes to structural changes in the lungs seen in pulmonary hypertension, asthma, and pulmonary fibrosis conditions commonly observed in SCD. Elevated levels of proline, a by-product of arginase activity, are associated with pulmonary hypertension and may contribute to fibrosis and airway remodeling by aiding collagen synthesis. Higher ornithine levels may also stimulate polyamine production, necessary for cell growth seen in vascular remodeling. Asthma involves inflammation-driven increases in arginase activity, causing acute shortages of arginine and NO. Acute chest syndrome and various forms of pulmonary hypertension also relate to acute shortages of these substances. It is noteworthy that asthma significantly raises the risk of ACS, suggesting a possible interaction between these conditions. Moreover, both asthma and ACS are tied to a specific NO synthase gene variation [34]. 

### 4.2. Overlap in Inflammatory Pathways between Hemoglobinopathies and Asthma

Research by Newaskar et al. indicates that SCD is a pro-inflammatory condition with elevated levels of serum inflammatory markers, which further increase during VOC and ACS [35]. In a review by Samarasinghe et al., it was observed that the co-existence of asthma and SCD raises significant questions about how these two conditions interact, given that the inflammatory pathways associated with both SCD and asthma are intricate and exhibit some overlap. Research suggests that endothelial activation is a significant pathway through which sickled red blood cells contribute to vaso-occlusion, initiating injury via reactive oxygen species and activating endothelial cells. This activation leads to the infiltration of other cells such as monocytes and neutrophils, contributing to uncontrolled cell adhesion in blood vessels. Increased levels of pro-inflammatory cytokines have also been noted in SCD patients, contributing to multiple complications, including pulmonary dysfunction and susceptibility to infections. Both asthma and SCD involve airway inflammation, and there is an overlap in the inflammatory pathways between the two conditions. This shared inflammation may contribute to an asthma-like phenotype in SCD patients or increase the likelihood of asthma development in this population. Moreover, existing models of asthma in SCD mice suggest that these mice respond more severely to allergen exposures, with heightened airway inflammation and pathology [36]. According to research by Nithichanon et al., individuals with beta-thalassemia are more susceptible to bacterial infections, particularly those caused by Burkholderia pseudomallei (Bp), which causes melioidosis in Thailand [37]. This increased vulnerability could be caused by a fundamental innate antiviral immune failure associated with epithelial barrier fragility or deficiencies in TLR7 or interferon production. The production of heme oxygenase 1 (HO-1), a putative regulator of immune function, was increased in beta-thalassemia patients’ blood both with and without Bp stimulation in vitro, highlighting compromised production of IFN-gamma and IL-10 in the blood of patients stimulated with bacteria. These results suggest that excess heme in beta-thalassemia significantly causes immune suppression through HO-1, contributing to susceptibility to severe bacterial infections [37]. The reduction in these cytokines could explain the baseline inflammatory state in thalassemic patients, also involving bronchial inflammation [38]. Furthermore, in some asthma endotypes, especially those with lower plasma levels of anti-inflammatory cytokines like IL-10, virus-induced exacerbations can still be a major concern even when the illness is properly controlled. In addition to having lower levels of IL-10, asthmatic thalassemic patients may also be more vulnerable to viruses by nature. This vulnerability may arise from a primary defect in innate antiviral immunity associated with the fragility and rupture of the epithelial barrier or from abnormalities in the production of TLR7 or interferon [3,38]. It seems that thalassemia and SCD patients have dysregulated inflammatory pathways, showing decreased levels of some cytokines and elevated levels of others. A study by Pierrot-Gallo et al. found that pro-inflammatory cytokines like IL-1β, IL-6, IL-8, and TNF-α are elevated in people with SCD, which may be linked to chronic endothelial activation and sickle cell and neutrophil adhesion to blood vessel endothelium [39]. Studies are currently being conducted to determine the exact mechanism by which these patients have higher amounts of some cytokines than others. This cytokine change may be the cause of these people’s heightened vulnerability to asthma since IL-6 plays a role in non-TH2-mediated asthma processes [40]. 

### 4.3. Anemia as a Risk Factor for Asthma

According to the study by Eissa et al., which aligns with the findings of Ramakrishnan et al., anemia itself emerges as another risk factor for pediatric asthma [41,42]. Two hundred children with upper or lower respiratory tract infections, aged two to eighteen, participated in their study. A control group of 100 age- and sex-matched children without anemia was selected, and 100 anemic youngsters comprised the study group. According to this research, children who were anemic were 5.75 times more likely to experience an asthma attack than children who were not anemic. They suggested that the higher frequency of asthmatic episodes in anemic children might be due to the following reasons: hemoglobin plays a crucial role in transporting oxygen and carbon dioxide, acting as a buffer and inactivating nitric oxide. Hemoglobin is the main factor that regulates oxygen pressure in tissues. A decrease in hemoglobin, whether in quality or quantity, can negatively impact normal bodily functions [41,42].

## 5. Discussion

**Figure 1:**
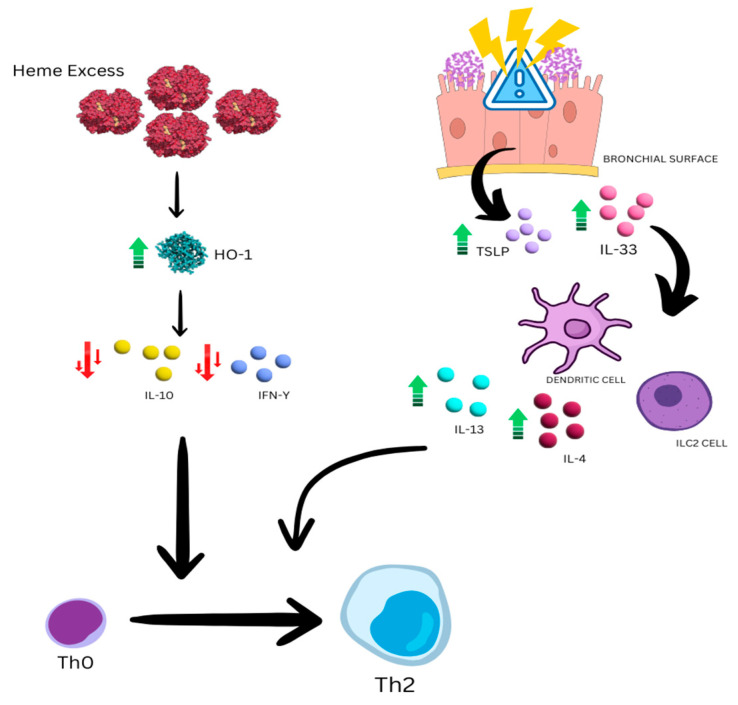
Stress and immune dynamics in pulmonary epithelium. Physical stress and bacterial and viral infections are sources of stress for the pulmonary epithelium, which responds by secreting TSLP and IL33. These stimulate dendritic cells and ILC2 cells to produce IL4 and IL13. On the other hand, the excess of heme in thalassemic patients causes an increase in the activity of heme oxygenase-1, leading to a decrease in the production of IL-10 and IFN-Y. These factors favor the transition from T0 to Th2 with the creation of a proallergenic phenotype.

The ongoing debate revolves around whether the presence of “asthma” in the context of hemoglobinopathies is primarily influenced by genetic and environmental factors or is instead a result of the underlying inflammatory and hemolytic condition. Recent studies indicate that asthma occurs in 20 to 48% of children with SCD, with a higher prevalence in older children [26]. The presence of atopy is a strong predictor of asthma in SCD, affecting up to 50% of affected children [29]. A systematic review by DeBaun et al. noted that asthma is common among children with sickle-cell disease, leading to heightened rates of severe vaso-occlusive pain and acute chest syndrome episodes. The presence of sickle-cell-associated lung disease, elevated IgE levels, and clinical manifestations resembling asthma underscore the importance of routinely evaluating asthma risk factors and symptoms during clinical visits. Spirometry should be used alongside respiratory history to detect and monitor lower airway obstruction and treatment response, given the significant prevalence of both asthma and non-asthmatic airway hyper-responsiveness in this population [43]. Comorbid asthma in SCD patients is associated with a higher mortality risk, as evidenced by various studies. Wheezing and shortness of breath are common symptoms, with a history of wheezing linked to an increased risk of ACS [43]. It was highlighted that the symptoms of asthma in SCD are caused by bronchial hyperresponsiveness, episodic bronchoconstriction, and acute-on-chronic inflammation. These exacerbations lead to mucous plugging, ventilation–perfusion mismatch, and hypoxemia, which can trigger the sickling of red blood cells and predispose individuals to ACS [34,44,45]. The interplay of inflammatory pathways in conditions such as SCD and thalassemia underscores the complexity of respiratory health in affected individuals. Dysregulated cytokine profiles, endothelial dysfunction, and immune activation contribute to the pathophysiology of asthma and related respiratory complications in hematologic disorders (Figure 1). 

**Figure 2:**
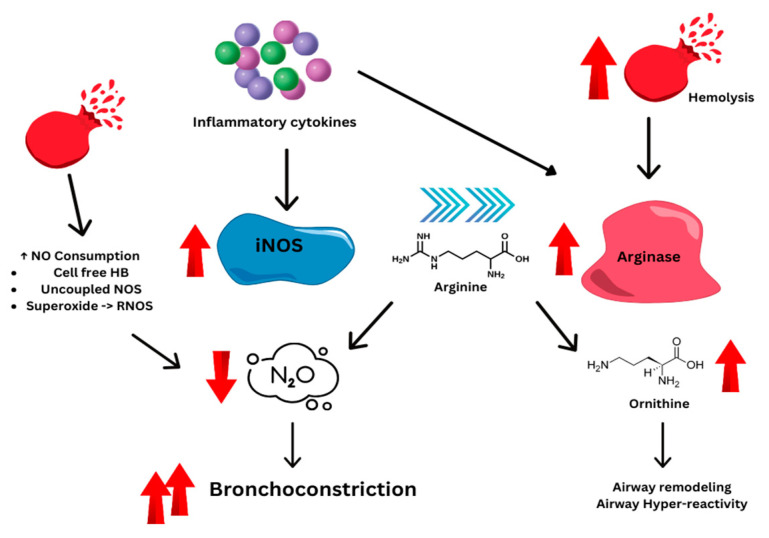
Novel paradigm of hemolysis-associated AHR and an “asthma-like” condition in SCD. The image presents a biochemical pathway illustrating the impact of dysregulated arginine metabolism on pulmonary health. We show how hemolysis leads to an increase in arginase activity, shifting the metabolism of arginine away from the production of nitric oxide (NO). This metabolic shift increases ornithine, which contributes to lung structure changes associated with conditions like pulmonary hypertension, asthma, and pulmonary fibrosis, which are prevalent in SCD patients. The image highlights the conversion of arginine to ornithine, which is linked to pulmonary hypertension and possible fibrosis and how its increment may promote cell growth and vascular remodeling. Additionally, it outlines the role of inflammation in enhancing arginase activity during asthma attacks, which can result in acute arginine and NO deficiency.

Studies by Morris et al. and Samarasinghe et al. highlight the overlapping inflammatory pathways between asthma and SCD, suggesting shared mechanisms of airway inflammation and immune dysregulation [34,35,36]. It has been found that arginine deficiency plays a significant role in the pathophysiology of both SCD and allergic asthma. The reduced availability of arginine is associated with various complications in SCD, such as pulmonary hypertension and VOC. Studies using murine models have demonstrated that arginine deficiency in nitric oxide synthase leads to decreased levels of nitric oxide, contributing to increased airway hyperresponsiveness in asthma. In SCD, heightened hemolysis results in the release of cellular arginase, further diminishing arginine reserves, in particular during vaso-occlusive crises [34,36]. In individuals with both SCD and asthma, heightened inflammation may result in increased expression of arginase and inducible nitric oxide synthase, worsening arginine depletion (Figure 2). 

Integrating screening protocols into routine clinical practice can improve outcomes and reduce the burden of respiratory complications in hematologic disorders [32]. Investigations into the immunological consequences of SCD and thalassemia reveal potential vulnerabilities to viral infections and bacterial complications, further complicating respiratory health in affected individuals. The role of proinflammatory cytokines, such as IL-6, in modulating immune responses and exacerbating asthma-like symptoms underscores the importance of understanding the systemic effects of hematologic disorders on respiratory function. The findings discussed have important clinical implications for the diagnosis and management of asthma in individuals with hematologic disorders. Screening protocols, such as those proposed by Duckworth et al. and Sadreameli et al., offer valuable strategies for identifying asthma and pulmonary function abnormalities in pediatric SCD patients, facilitating early intervention and targeted treatment [23,31]. Additionally, studies examining the utility of screening tools, such as the BCIS, in preschool-aged children with SCD highlight the importance of the early detection and management of asthma in vulnerable populations [32]. Collaboration between pulmonologists, hematologists, and other healthcare professionals is crucial for optimizing care for these complex cases. Future research should focus on elucidating the underlying mechanisms of asthma in SCD and thalassemia, including the role of inflammatory pathways, immune dysregulation, and genetic predispositions. A next step forward could be to investigate targeted treatments for patients suffering from both hemoglobinopathies and asthma. While there are no FDA-approved therapies specifically for the interplay between asthma and hemoglobinopathies, current treatments for each condition can be used to manage patients with both diseases. Additionally, new and interesting results may arise from patients using biologic drugs targeting various interleukins, such as dupilumab and tezepelumab, to determine if the anti-inflammatory action of these drugs can also help reduce ACS or VOC events in patients with asthma and SCD [46,47].

## 6. Conclusions

The association between asthma and major hemoglobinopathies, including thalassemia and SCD, reveals a complex interplay between these conditions. Studies demonstrate a significant correlation between thalassemia and increased vulnerability to asthma, particularly in children. Similarly, individuals with SCD exhibit a heightened risk of asthma-like symptoms, contributing to increased morbidity and mortality. The underlying mechanisms involve dysregulated inflammatory pathways and shared immunological consequences, suggesting overlapping pathophysiological mechanisms. Moreover, asthma diagnosis in individuals with SCD may act as an early indicator of a higher risk of ACS. Despite the prevalence of asthma in major hemoglobinopathies, the underlying mechanisms driving its development and immunological features remain poorly understood, posing challenges for effective asthma management in these patients. Further research is essential to fully elucidate the intricate relationship between asthma and hemoglobinopathies, paving the way for tailored treatment approaches that address the unique needs of patients with coexisting conditions. Additionally, screening protocols for asthma among individuals with hemoglobinopathies, particularly in pediatric populations, could improve early detection and management, thereby reducing the burden of respiratory complications associated with these disorders.

## Figures and Tables

**Table 1:** Asthma and thalassemia.

Reference	Study Design	Objectives	Population	Methods	Results
Hsieh, 2021 [19]	Retrospective cohort study.	Investigated the association between thalassemia and asthma in children. Determined the risk of developing asthma in children with thalassemia.	One million individuals were randomly selected from the Registry for Beneficiaries of the National Health Insurance Research Database (born between 1997–2010), from which were selected 800 thalassemic children matched with 3200 non-thalassemic control children.	Thalassemic children were matched with control children without thalassemia based on sex, birth year, birth season, prematurity, and previous enteroviral infection. Incidence rates of asthma were compared.	Children with thalassemia had higher rates of developing asthma than the non-thalassemia controls with an adjusted hazard ratio of 1.37 ([CI 95%] = 1.19–1.58). Boys in the thalassemia cohort had a significantly higher (aIRR) of asthma than those in the non-thalassemia cohort (adjusted IRR = 1.45, 95% CI = 1.02–1.73). The risk of atopic and nonatopic asthma was higher in the thalassemia cohort than in the non-thalassemia cohort (IRR = 1.3, 1.61, respectively).
Rhew, 2019 [20]	Cross-sectional study.	The objective of the study was to determine whether inflammatory atopic diseases (atopic dermatitis, allergic rhinitis, and asthma) were associated with anemia in pediatric patients.	The study included a total of 846,718 pediatric patients from HIRA’s (Health Insurance Review and Assessment Service) dataset of South Korea in 2016.	The study analyzed the association between atopic disease and iron deficiency anemia (IDA).	The study found that there was an association between atopic diseases and IDA in pediatric patients. The adjusted odds ratio (aOR) of IDA was 1.42 for atopic dermatitis, 1.25 for allergic rhinitis, and 1.71 for asthma. IDA was more prevalent in patients with multiple comorbid atopic diseases, with aOR of 1.30 for 1 atopic diagnosis, 1.81 for 2 atopic diagnoses, and 2.58 for 3 atopic diagnoses.
Palma-Carlos, 2005 [21]	Retrospective cohort study.	Evaluated the incidence of asthma in patients with hemoglobinopathies allergic to house dust mites.	The study included 63 patients with hemoglobinopathies and allergy to dust mites.	66 hemoglobinopathy patients were compared to 491 respiratory allergic patients without hemoglobinopathies.	The prevalence of asthma was significantly higher in patients with thalassemia minor and sickle cell traits compared to the control group.

## Data Availability

Not applicable.
